# Sleep Deprivation Promotes Colorectal Cancer in Apc^Min/+^ Mice Associated with Modulation of Gut Microbiota and Related Metabolites

**DOI:** 10.7150/ijbs.134241

**Published:** 2026-05-29

**Authors:** Jia Chen, Rishun Su, Yong Kuang, Xiumin Liu, Yulong He, Jiong Chen, Changhua Zhang

**Affiliations:** 1 Digestive Diseases Center, The Seventh Affiliated Hospital of Sun Yat-sen University, Shenzhen, Guangdong, 518107, China.; 2 Guangdong Provincial Key Laboratory of Digestive Cancer Research, The Seventh Affiliated Hospital of Sun Yat-sen University, Shenzhen, Guangdong, 518107, China.; 3 Department of Anesthesiology, The Seventh Affiliated Hospital of Sun Yat-sen University, Shenzhen, Guangdong, 518107, China.

**Keywords:** sleep deprivation, colorectal cancer, gut microbiota, taurocholic acid

## Abstract

Sleep deprivation (SD) has emerged as an important environmental factor associated with colorectal cancer (CRC); however, the underlying mechanisms, particularly those involving the gut microbiota-metabolite axis, remain poorly understood. In this study, Apc^Min/+^ mice, a well-established CRC model, were subjected to SD using a modified multiple-platform method. Fecal samples were analyzed using 16S rRNA gene sequencing and untargeted metabolomics, and tumor burden, intestinal inflammation, and gut barrier integrity were assessed by hematoxylin and eosin staining, immunofluorescence, and Alcian blue-periodic acid-Schiff staining. Our results showed that SD significantly aggravated CRC progression, as evidenced by an increase in tumor number and severity, impaired intestinal barrier integrity, and enhanced intestinal inflammation. In parallel, SD markedly altered the gut microbiota composition, characterized by a pronounced reduction in beneficial bacteria such as Lactobacillus. Metabolomic profiling revealed significant metabolic alterations, with taurocholic acid (TCA) identified as a prominently elevated metabolite. Transcriptomic and functional analyses suggested that TCA may be involved in CRC progression through the activation of the MAPK/ERK signaling pathway. Collectively, these findings support a model in which SD contributes to CRC progression in association with alterations in the gut microbiota and related metabolites.

## Introduction

Colorectal cancer (CRC) is one of the most prevalent malignancies worldwide and one of the principal causes of cancer-related mortality 1. CRC imposes a sustained and substantial disease burden worldwide. Accumulating evidence indicates that this burden is closely associated with lifestyle changes that accompany socioeconomic development, including alterations in dietary patterns, tobacco use, and sleep-related behaviors 2-8. Accumulating epidemiological evidence indicates that sleep deprivation (SD) is positively associated with the development of multiple cancer types, including CRC, hepatocellular carcinoma, prostate cancer, and lymphoma 9-13. Human studies suggest that SD correlates with a higher incidence and mortality rate of CRC 14, 15, whereas experimental investigations further indicate that disruption of circadian rhythmicity facilitates CRC metastasis in animal models 16. However, the mechanisms by which SD contributes to CRC progression remain largely unknown.

Recent studies showed that SD alters gut microbial diversity 17, 18 and established a close association between dysbiosis of the gut microbiota and CRC. Although accumulating evidence suggests that SD can induce gut microbiota dysbiosis and contribute to CRC progression, the specific microbiota-associated metabolites and downstream signaling pathways linking SD-induced microbial alterations to CRC development remain largely unclear.

**Figure 1:**
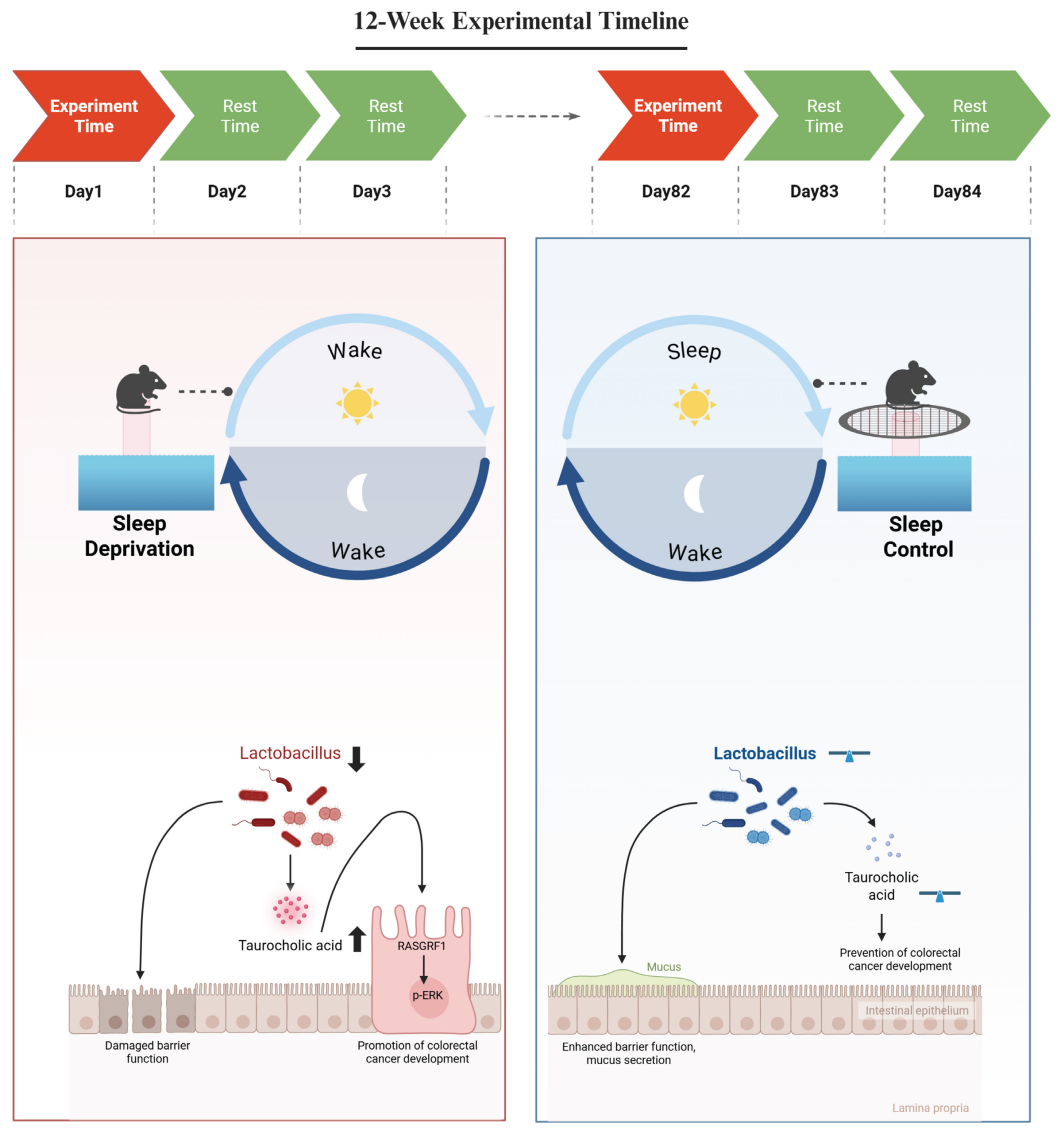
Mechanistic Analysis of Sleep Deprivation-Induced Colorectal Cancer Progression in Apc^Min/+^ Mice. Sleep deprivation reduces the abundance of Lactobacillus and increases intestinal taurocholic acid levels. Taurocholic acid promotes the development of colorectal cancer by upregulating RasGRF1 expression and activating the p-ERK signaling pathway. Figure created in BioRender. https://BioRender.com.

In the present study, we investigated whether SD-associated gut microbiota remodeling is linked to CRC progression through specific microbial-derived metabolites and downstream signaling pathways. Using the Apc^Min/+^ mice model, we found that SD disrupted gut microbial homeostasis, which was accompanied by pronounced alterations in microbial metabolite profiles. Among the altered metabolites, taurocholic acid (TCA) was selected for further analysis because it was markedly elevated, enriched in bile acid-related metabolic pathways, and was associated with SD-related microbial alterations. We further observed that elevated TCA levels were associated with activation of the MAPK/ERK signaling pathway in CRC cells, supporting a model in which SD-associated microbiota and metabolite alterations may contribute to CRC progression (Figure 1).

## Methods

### Mouse Colorectal Cancer Model and Sleep Deprivation

Male Apc^Min/+^ mice with a C57BL/6 background were used as models of spontaneous CRC driven by genetic mutations. Eight-week-old mice were randomly allocated to two experimental groups, the SD and the sleep control (SC) groups, with six animals in each group. SD was induced using a modified multiplatform paradigm. Briefly, several small circular platforms were fixed to the bottom of a water tank, after which water was added to maintain the water level slightly below the upper surface of the platforms. Mice in the SD group were placed on these platforms. Owing to the rodents' innate avoidance of water, the animals remained on the platforms. Upon falling asleep, reduced muscle tone caused them to lose balance and contact the water, resulting in immediate arousal, after which they returned to the platforms. The repetition of this process effectively disrupted continuous sleep. In contrast, the mice in the SC group were housed under identical environmental conditions, except that the platforms were overlaid with a wire mesh, enabling normal sleep without the risk of water contact. Both the SD and SC groups were maintained under the same light-dark cycle, ambient temperature, and general housing conditions, with the only difference being the SD setup.

The SD regimen consisted of a 24-h SD period followed by a 48-h recovery period, and this cycle was repeated over a total duration of 12 weeks. This experimental schedule was designed with reference to prior studies linking SD to CRC; however, most existing models have relied on relatively short-term SD exposure. In contrast, SD in humans typically manifests as a chronic condition and intestinal tumorigenesis is a gradual and cumulative process. Consequently, short-duration SD protocols may be insufficient for recapitulating the long-term effects observed in humans. To accurately model chronic SD and its influence on CRC development, a 12-week SD protocol was adopted in the present study.

Throughout the experimental period, the animals in both the SD and SC groups had unrestricted access to standard chow and water. At the end of the 12-week protocol, tissue specimens were harvested from Apc^Min/+^ mice for subsequent analyses. Tumor counting, histological evaluation, and image quantification were performed in a blinded manner. All animal experiments were reviewed and approved by the Institutional Animal Care and Use Committee and performed in compliance with established ethical standards and national guidelines governing the use of laboratory animals.

### 16S rRNA Sequencing of Mouse Fecal Samples

Total genomic DNA was extracted from fecal samples collected from both the SD and SC groups using the E.Z.N.A.® Soil DNA Kit (Omega Bio-tek, Norcross, GA, USA), following the manufacturer's instructions. The integrity of the extracted DNA was assessed using 1% agarose gel electrophoresis, and the DNA concentration and purity were determined using a NanoDrop 2000 spectrophotometer (Thermo Scientific, USA).

The V3-V4 hypervariable regions of the 16S rRNA gene were amplified by polymerase chain reaction (PCR) using barcode-indexed primers 338F (5'-ACTCCTACGGGAGGCAGCAG-3') and 806R (5'-GGACTACHVGGGTWTCTAAT-3'), with the extracted DNA serving as the template. The PCR products from the same sample were pooled and subjected to 2% agarose gel electrophoresis for size verification and purification of the target amplicons. Purified PCR products were quantified using a Synergy HTX microplate reader (BioTek, USA).

Sequencing libraries were generated using the NEXTFLEX Rapid DNA-Seq Kit (Bio Scientific, USA), following the manufacturer's recommended procedures. High-throughput sequencing was subsequently performed on an Illumina NextSeq 2000 system, employing a paired-end strategy with a read length of 300 bp at each end.

### High-throughput Sequencing Data Analysis

Raw paired-end sequencing reads were initially processed using FASTP (version 0.19.6) for quality filtering and trimming. Forward and reverse reads that passed quality control were merged into contiguous sequences using FLASH (version 1.2.11). Sample demultiplexing and read orientation were achieved by identifying the barcode and primer sequences embedded within the reads, followed by strand correction according to specific barcode-primer configurations.

Amplicon sequence variants (ASVs) were inferred by denoising the sequencing data using the QIIME2 workflow. To standardize the sequencing depth among the samples, the resulting feature table was rarefied to 20,000 reads per sample. The taxonomic assignment of the ASVs was subsequently performed using the SILVA reference database for 16S rRNA genes (version 138).

### LC-MS/MS Analysis

Liquid chromatography-tandem mass spectrometry (LC-MS/MS) was performed using a Thermo UHPLC-Q Exactive HF-X system with technical support provided by Shanghai Majorbio Bio-pharm Technology Co., Ltd. Chromatographic separation was achieved on an HSS T3 column (2.1 mm × 100 mm, 1.8 µm particle size). The mobile phase system was configured as follows: mobile phase A consisted of water/acetonitrile (95:5, v/v) with 0.1% formic acid and mobile phase B consisted of acetonitrile/isopropanol/water (47.5:47.5:5, v/v/v) with 0.1% formic acid. The flow rate was maintained at 0.40 mL/min, and the column temperature was set at 40°C.

Mass spectrometric data were acquired in both positive and negative ion modes, with a mass scan range of 70-1,050 m/z. The sheath gas flow rate was set to 50 psi, and the auxiliary gas flow rate was maintained at 13 psi, with the auxiliary gas heater temperature at 425°C. The electrospray ionization voltage was 3,500 V in both positive and negative modes, and the ion transfer tube temperature was set at 325°C. Normalized collision energies of 20, 40, and 60 V were applied in stepped cyclic mode. The resolution was set to 60,000 for MS1 and 7,500 for MS2. Data were acquired in data-dependent acquisition (DDA) mode.

### Metabolite Identification and Analysis

Raw LC-MS data were processed using the Progenesis QI software, which included key steps such as chromatographic peak detection and retention time alignment. Metabolites were identified by matching spectral features against the Human Metabolome Database (HMDB), Metlin, and an in-house reference database. Data preprocessing involved applying the 80% rule for missing values, sum normalization, and quality control (QC) filtering (with RSD < 30%). Multivariate statistical analyses were conducted using the ropls package in R. Differential metabolites were identified based on variable importance in projection (VIP) scores greater than one from orthogonal partial least squares-discriminant analysis (OPLS-DA), combined with Student's t-test results (p < 0.05).

### RNA Sequencing (RNA-seq)

Total RNA was extracted from cells in the TCA and control groups using the TRIzol reagent (Invitrogen) according to the manufacturer's instructions. Library preparation and RNA sequencing were performed by Tsingke Biotechnology (Beijing, China). Messenger RNA (mRNA) was purified using oligo(dT)-conjugated magnetic beads, followed by first- and second-strand cDNA synthesis through reverse transcription to obtain double-stranded cDNA. The final libraries were amplified via PCR to generate DNA nanoballs (DNBs), which were then loaded into the patterned nanoarrays. Sequencing was performed using a BGISEQ-500 platform. Gene expression levels were quantified using StringTie, and differential expression analysis was conducted using DESeq2. Genes with Q-values ≤ 0.05 were considered significantly differentially expressed. Kyoto Encyclopedia of Genes and Genomes (KEGG) pathway enrichment analysis was performed using the Phyper function.

### Statistical Analysis

Statistical analyses were performed using GraphPad Prism software (version 9.0; GraphPad Software, La Jolla, CA). Appropriate statistical tests were selected for intergroup comparisons based on the distribution characteristics of the variables. For variables that conformed to the assumption of normality, an unpaired Student's t-test was applied. The Wilcoxon rank-sum test was used for variables that did not meet normality assumptions. Categorical variables between groups were compared using the chi-squared (*χ*^2^) test. A p-value of < 0.05 was considered statistically significant.

Six mice were included in each group for animal experiments. For downstream analyses, a subset of animals was used depending on tissue availability and assay requirements. The exact sample size for each assay is indicated in the corresponding figure legend, and the plotted data points represent biological replicates unless otherwise stated.

## Results

### SD Promotes the Progression of CRC in Apc^Min/+^ Mice

**Figure 2:**
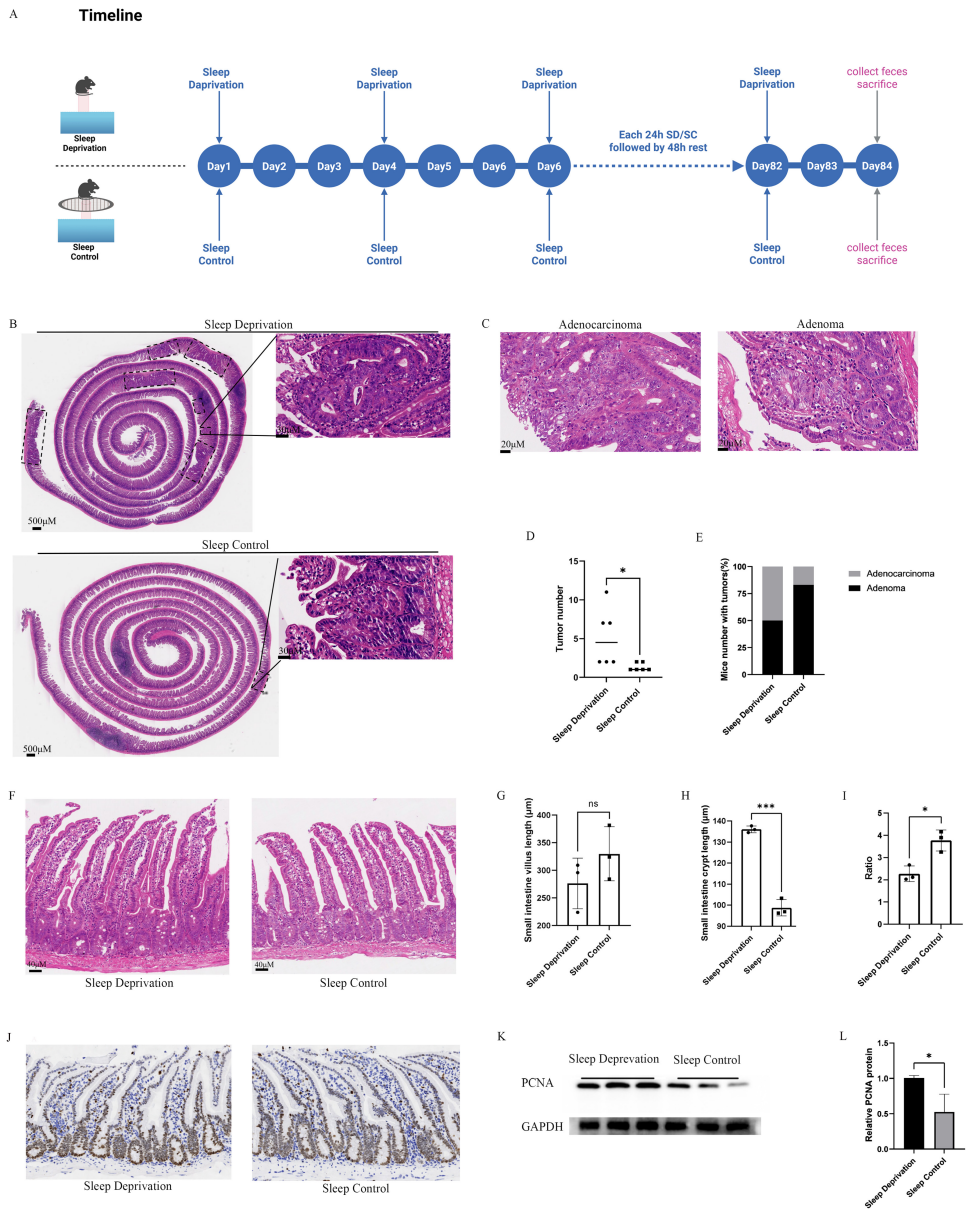
Effects of Sleep Deprivation on Intestinal Tumor Number and Malignancy in Mice. A. Schematic representation of the sleep deprivation protocol: 24 h deprivation followed by 48 h rest, repeated for 12 weeks. B. Representative HE-stained image showing intestinal tumor counts in the SD and SC groups; each dashed box indicates an individual intestinal tumor. C. Representative HE-stained image of intestinal adenocarcinoma and intestinal adenoma. D. Quantification of intestinal tumor numbers in the SD and SC groups (n = 6 mice/group). E. Proportion of mice with malignant transformation of intestinal tumors in SD and SC groups. F. Representative images of intestinal villi and crypts in the small intestine of SD and SC groups. G. Statistical comparison of small intestinal villus length between SD and SC groups (n = 3 mice/group). H. Statistical comparison of small intestinal crypt depth between SD and SC groups (n = 3 mice/group). I. Statistical comparison of villus-to-crypt ratio in the small intestine between SD and SC groups (n = 3 mice/group). J. Immunohistochemical staining of Ki67 in intestinal tissue sections from the SD and SC groups. K. Western blot analysis of PCNA in intestinal tissues of mice (n = 3 mice/group). L. Quantification of relative PCNA expression levels in intestinal tissues of mice (n = 3 per group).

To assess the effect of SD on CRC progression, Apc^Min/+^ mice were randomly divided into SD and SC groups with six mice in each group. The SD protocol consisted of repeated cycles of 24-h deprivation followed by a 48-h recovery over a 12-week period (Figure 2A). Compared with the SC group, the SD group developed a significantly greater tumor burden and displayed a higher frequency of malignant lesions, indicating an overall enhancement in CRC progression (Figures 2B-E).

Histological analysis revealed a tendency toward reduced intestinal villus length in the SD group compared with the SC group; although this difference was not statistically significant (Figures 2F and 2G). In contrast, crypt depth was markedly increased in the SD group relative to that in the SC group (Figure 2H). Consistently, the villus-to-crypt ratio was significantly decreased in the SD group (Figure 2I). Collectively, these morphological alterations indicate that SD is associated with structural disruption of the intestinal epithelium.

In addition, the SD group exhibited a pronounced increase in the proportion of Ki67-positive intestinal epithelial cells, accompanied by increased expression of proliferating cell nuclear antigen (PCNA), when compared to that in the SC group (Figures 2J-L).

### SD Disrupts Intestinal Barrier Function and Promotes Intestinal Inflammation in Apc^Min/+^ Mice

**Figure 3:**
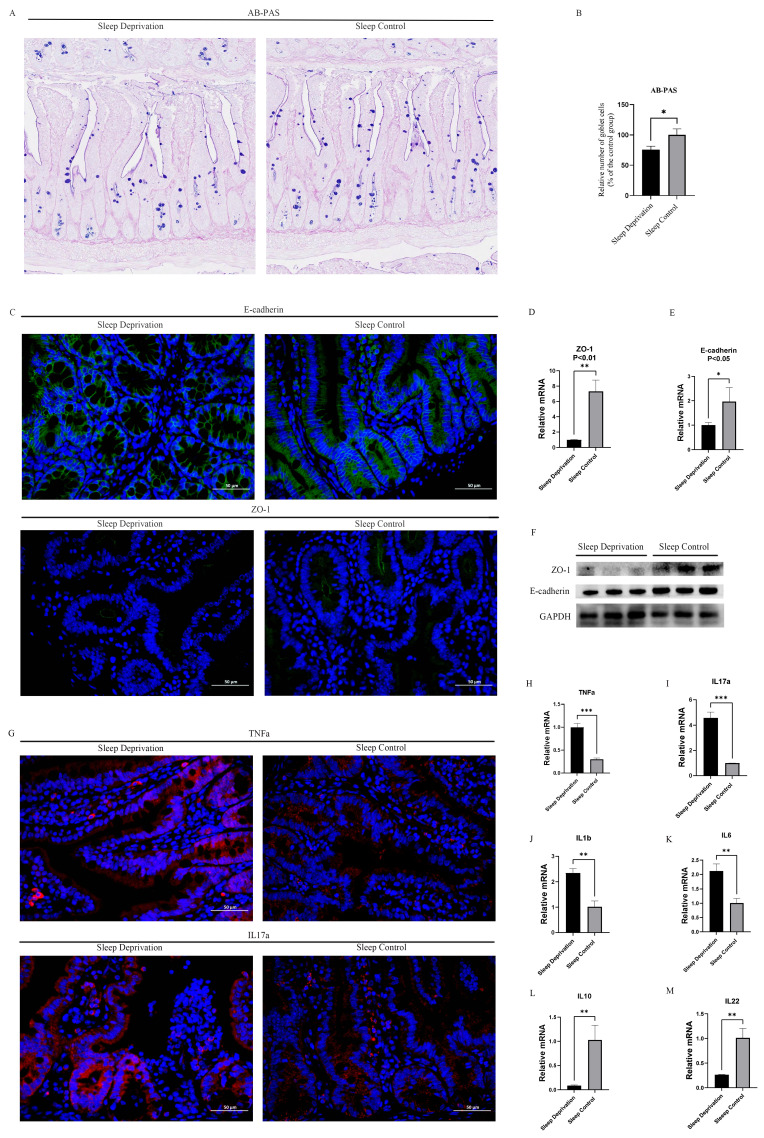
SD impairs intestinal barrier function and promotes intestinal inflammation in mice. A. Representative AB-PAS-stained sections of small intestinal tissue from SD and SC groups. B. Quantification of goblet cell numbers in the intestinal epithelium, showing a significant reduction in the SD group compared to the SC group. C. Representative immunofluorescence images of E-cadherin and ZO-1 staining in intestinal tissue sections from SD and SC groups; (D-E) RT-qPCR analysis of ZO-1 (*Tjp1*) and E-cadherin (*Cdh1*) expression in intestinal tissues. F. Western blot analysis of ZO-1 and E-cadherin in intestinal tissues. G. Representative immunofluorescence images of TNF-α and IL-17A in intestinal tissue sections from SD and SC groups; (H-M) RT-qPCR analysis of *Tnf, Il17a, Il1b, Il6, Il10*, and *Il22* expression in intestinal tissues. For quantitative analyses, n = 3 biological replicates per group. Representative images are shown. For image-based analyses, multiple representative fields were analyzed per mouse and averaged for statistical analysis. Western blot analysis was based on biological replicates.

The integrity of the intestinal barrier is essential for maintaining intestinal homeostasis, and impairment of this barrier is closely associated with CRC development. Goblet cell-derived mucins constitute a major component of the intestinal mucus layer and are critical for chemical barrier function. To evaluate goblet cell abundance, Alcian blue-periodic acid-Schiff (AB-PAS) staining was performed on paraffin-embedded intestinal sections. Quantitative analysis revealed a significant reduction in goblet cell numbers in the SD group compared with the SC group (Figures 3A and 3B). In parallel, immunofluorescence staining demonstrated decreased expression of the adherens junction protein E-cadherin and the tight junction-related protein zonula occludens-1 (ZO-1), in the predominantly non-neoplastic intestinal mucosa of the SD group (Figure 3C). Consistent with these findings, reverse transcription quantitative PCR (RT-qPCR) analysis showed significantly lower mRNA levels of ZO-1 (*Tjp1*) and E-cadherin (*Cdh1*) in the SD group than those in the SC group (p < 0.05; Figures 3D and 3E), which was further corroborated by western blot analysis (Figure 3F). Together, these data indicate a pronounced disruption in intestinal barrier integrity in response to SD.

Chronic inflammation is a well-known contributor to CRC. To evaluate inflammatory alterations in intestinal tissues, the expression of representative proinflammatory and anti-inflammatory mediators was assessed at both the protein and mRNA levels using immunofluorescence staining and RT-qPCR. Immunofluorescence analysis demonstrated markedly elevated levels of tumor necrosis factor-α (TNF-α) and interleukin-17A (IL-17A) in the intestines of the SD group compared to that in the SC group (Figure 3G). Consistent with these observations, RT-qPCR analysis revealed significant upregulation of multiple proinflammatory cytokines in the SD group, including *TNF-α*, *IL-17A*, *IL-1β*, and *IL-6* (Figures 3H-K). In contrast, the transcript levels of anti-inflammatory cytokines, such as *IL-10* and *IL-22*, were significantly reduced in the SD group relative to those in the SC group (Figures 3L and 3M). Collectively, these results indicated an enhanced inflammatory state in the intestinal tissues of Apc^Min/+^ mice subjected to SD.

### SD Alters the Composition of the Gut Microbiota in Apc^Min/+^ Mice

**Figure 4:**
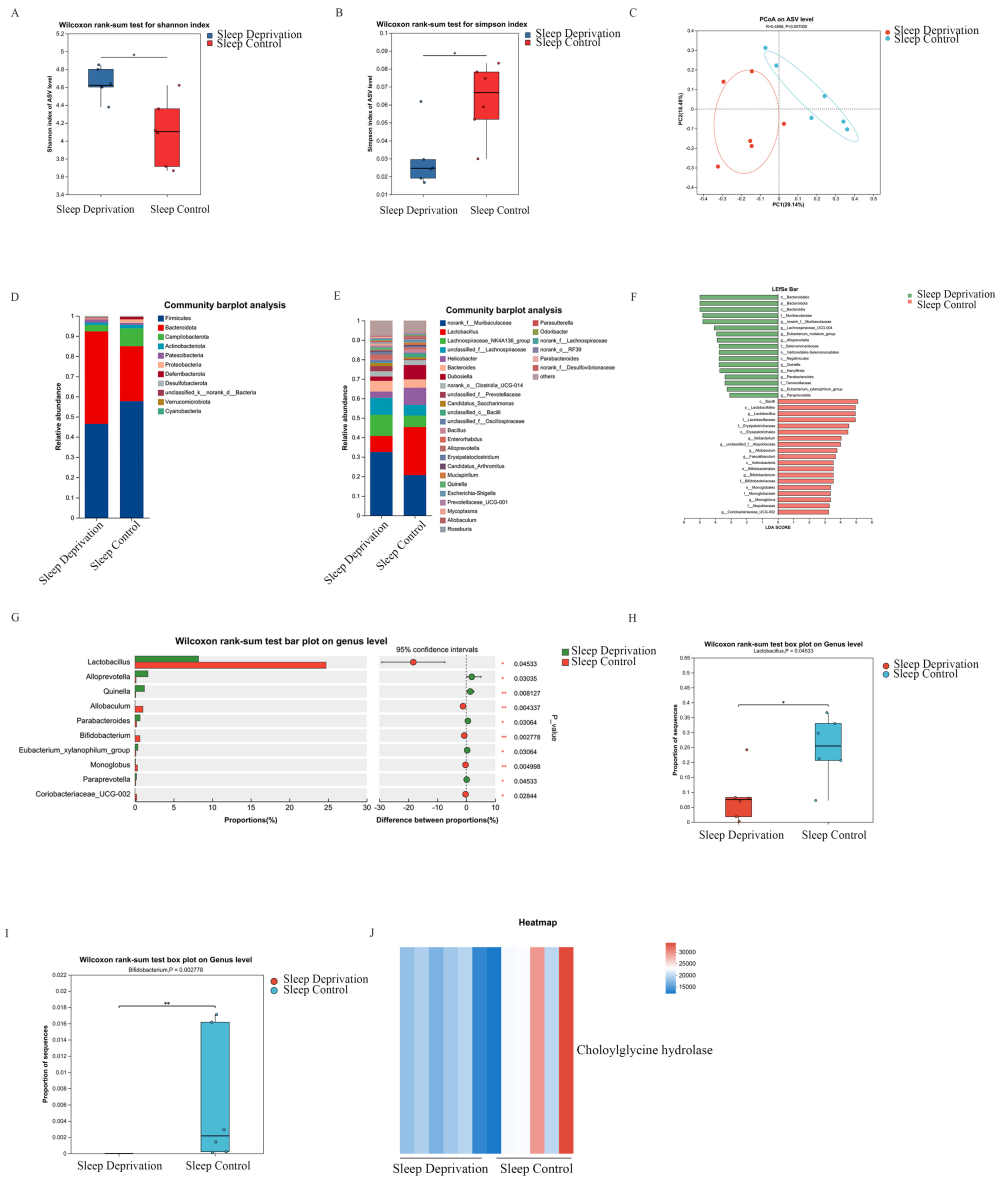
SD Alters the Composition of the Intestinal Microbiota in Apc^Min/+^ Mice. A. The Shannon index of the gut microbiota in the SD group was significantly higher compared with the SC group. B. The Simpson index of the gut microbiota in the SD group was significantly lower compared with the SC group. C. PCoA of gut microbiota in SD and SC groups. D. Bar chart of gut microbial community composition at the phylum level. E. Bar chart of gut microbial community composition at the genus level. F. LefSe analysis plot. G. Differential abundance analysis of gut microbiota at the genus level. H. Intergroup comparison of Lactobacillus. I. Intergroup comparison of Bifidobacterium. J. Heatmap showing the relative abundance of choloylglycine hydrolase between the SD group and the SC group.

To characterize the gut microbiota alterations associated with SD, fecal samples from SD and SC groups were subjected to 16S rRNA gene sequencing. Analysis of α-diversity showed a significant increase in the Shannon index accompanied by a significant decrease in the Simpson index in the SD group relative to that in the SC group (Figures 4A and 4B). In the present study, the original Simpson index was applied, with lower values indicating greater microbial diversity. Furthermore, β-diversity assessment using principal coordinates analysis (PCoA) revealed a clear separation between SD and SC groups, along with tight clustering within each group (Figure 4C), suggesting that SD is associated with marked alterations in the intestinal microbial community structure.

At the phylum level, the SD group exhibited a significantly higher relative abundance of Bacteroidetes compared to that in the SC group (Figure 4D). At the genus level, several taxa, including norank_f_Muribaculaceae, Alloprevotella, Quinella, Parabacteroides, Eubacteriumxylanophilum_group, and Paraprevotella were significantly enriched in the SD group, whereas Lactobacillus, Allobaculum, Bifidobacterium, and Monoglobus were markedly depleted (Figure 4E). Notably, the decrease in Lactobacillus was the most pronounced among the altered genera, highlighting the potential association between this taxon and SD-related CRC development.

Linear discriminant analysis effect size (LEfSe) further identified the key taxa that distinguished between the SD and SC groups. At the genus level, Lactobacillus showed the highest discriminative power, with a linear discriminant analysis (LDA) score of 4.94 (P < 0.05), which exceeded that of the leading genus enriched in the SD group, g_norank_f_Muribaculaceae (LDA = 4.80, P < 0.05). These results indicated that Lactobacillus was a major contributor to the microbial compositional differences observed between SD and SC groups (Figure 4F).

Differential abundance analyses were performed to further validate the observed genus-level compositional shifts. Several genera were significantly enriched in the SD group, whereas Lactobacillus, Allobaculum, Bifidobacterium, and Monoglobus showed markedly reduced relative abundances compared to those in the SC group (Figures 4G-I).

Functional profiling of the gut microbiota was conducted using KEGG-based prediction and PICRUSt2 analysis. This analysis revealed a pronounced reduction in the predicted abundance of choloylglycine hydrolase in the SD group compared to that in the SC group (Figure 4J), suggesting a potential alteration in microbial pathways related to bile acid metabolism based on PICRUSt2 prediction.

### Sleep Deprivation Alters Fecal Metabolites in Apc^Min/+^ Mice

**Figure 5:**
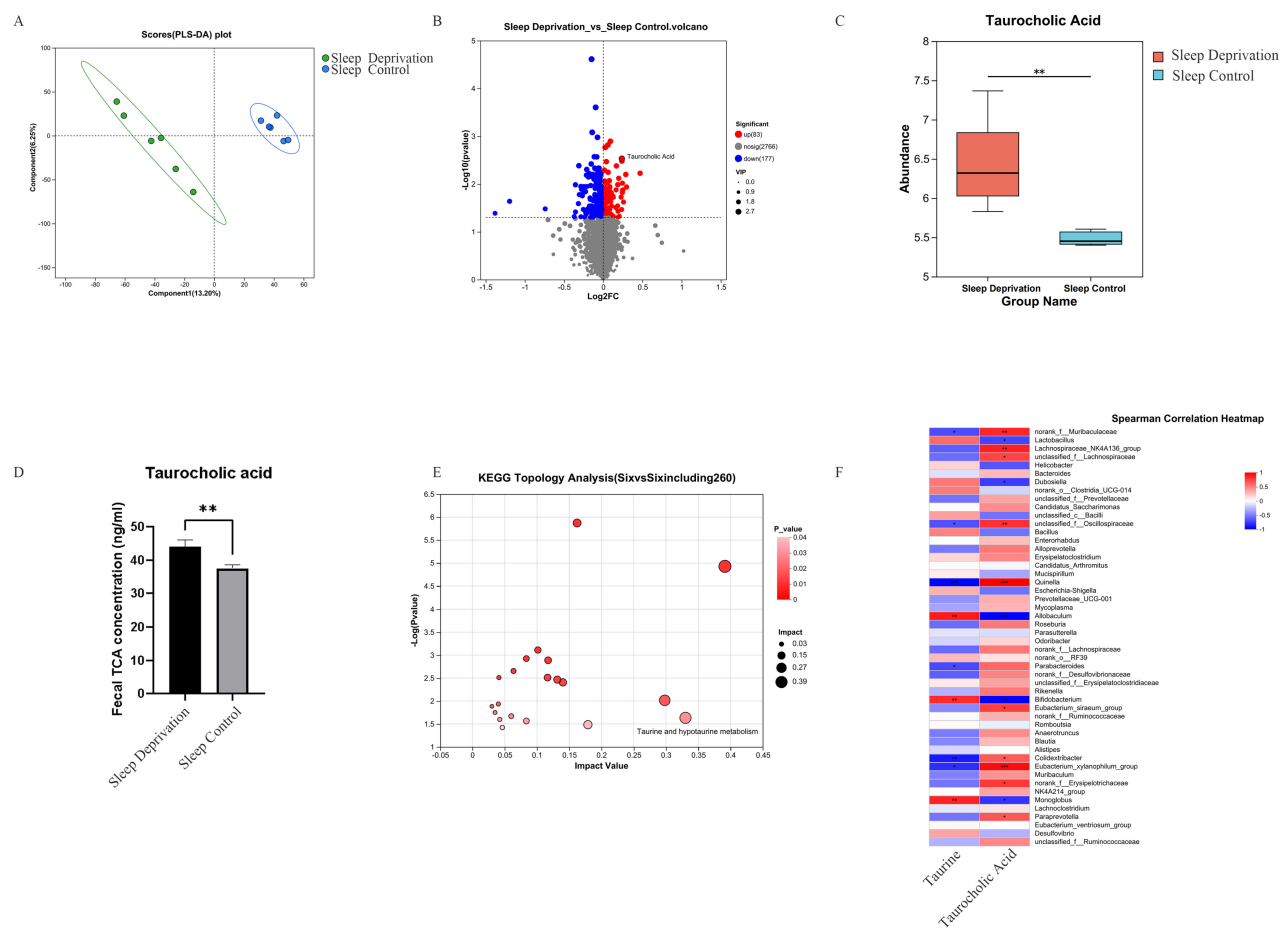
Sleep Deprivation Alters Fecal Metabolites in Apc^Min/+^ Mice. A. PLS-DA analysis of fecal metabolites in SD and SC groups; B. Volcano plot of differential metabolites; C. Intergroup differences in fecal TCA between SD and SC groups; D. Intergroup differences in fecal TCA validated by ELISA; E. Topological analysis of KEGG pathway enrichment for differential metabolites; F. Correlation analysis between gut microbiota and fecal differential metabolites in mice.

Given the regulatory role of microbiota-derived metabolites in intestinal physiology and pathology, an untargeted metabolomic analysis was conducted on fecal samples from SD and SC groups. Partial least squares-discriminant analysis (PLS-DA) revealed a clear separation between the metabolic profiles of the two groups, indicating substantial alterations in fecal metabolite composition following SD (Figure 5A). Differential metabolites were screened using a combination of variable importance in projection (VIP) values ≥ 1 derived from orthogonal PLS-DA (OPLS-DA) and Student's t-test results (p < 0.05), and their distribution was visualized using a volcano plot. In total, 260 metabolites exhibited significant differences between SD and SC groups, including 83 upregulated and 177 downregulated metabolites. Among these, TCA was one of the most prominently upregulated metabolites (Figures 5B and 5C). Consistent with the metabolomic findings, enzyme-linked immunosorbent assay (ELISA) validation confirmed a significant increase in fecal TCA levels in the SD group (Figure 5D). KEGG pathway enrichment analyses indicated that the TCA-related metabolic pathway—Taurine and hypotaurine metabolism—was among the most significantly enriched pathways (Figure 5E). Collectively, these data indicate that TCA is a key differential metabolite in sleep deprivation-induced metabolic changes.

### Correlation Analysis Between Gut Microbiota and Differential Fecal Metabolites in Apc^Min/+^ Mice

To examine the association between gut microbial alterations and fecal metabolites in Apc^Min/+^ mice, Spearman's correlation analysis was performed between the key differential metabolite TCA, its precursor taurine, and the top 50 bacterial genera ranked by relative abundance. The analysis revealed that several genera, including Lachnospiraceae_NK4A136_group, were significantly and positively correlated with TCA levels (p < 0.05). In contrast, Lactobacillus, Dubosiella, Allobaculum, and Bifidobacterium were significantly and negatively correlated with TCA abundance (p < 0.05). Notably, Lactobacillus, whose relative abundance was markedly reduced in the SD group, exhibited a strong inverse association with TCA levels (Figure 5F). Taken together, these results indicate a close relationship between SD-associated microbial shifts and alterations in bile acid-related metabolites.

### Taurocholic Acid Promotes Cell Proliferation and Exacerbates Impairment of Cell Junctions

**Figure 6:**
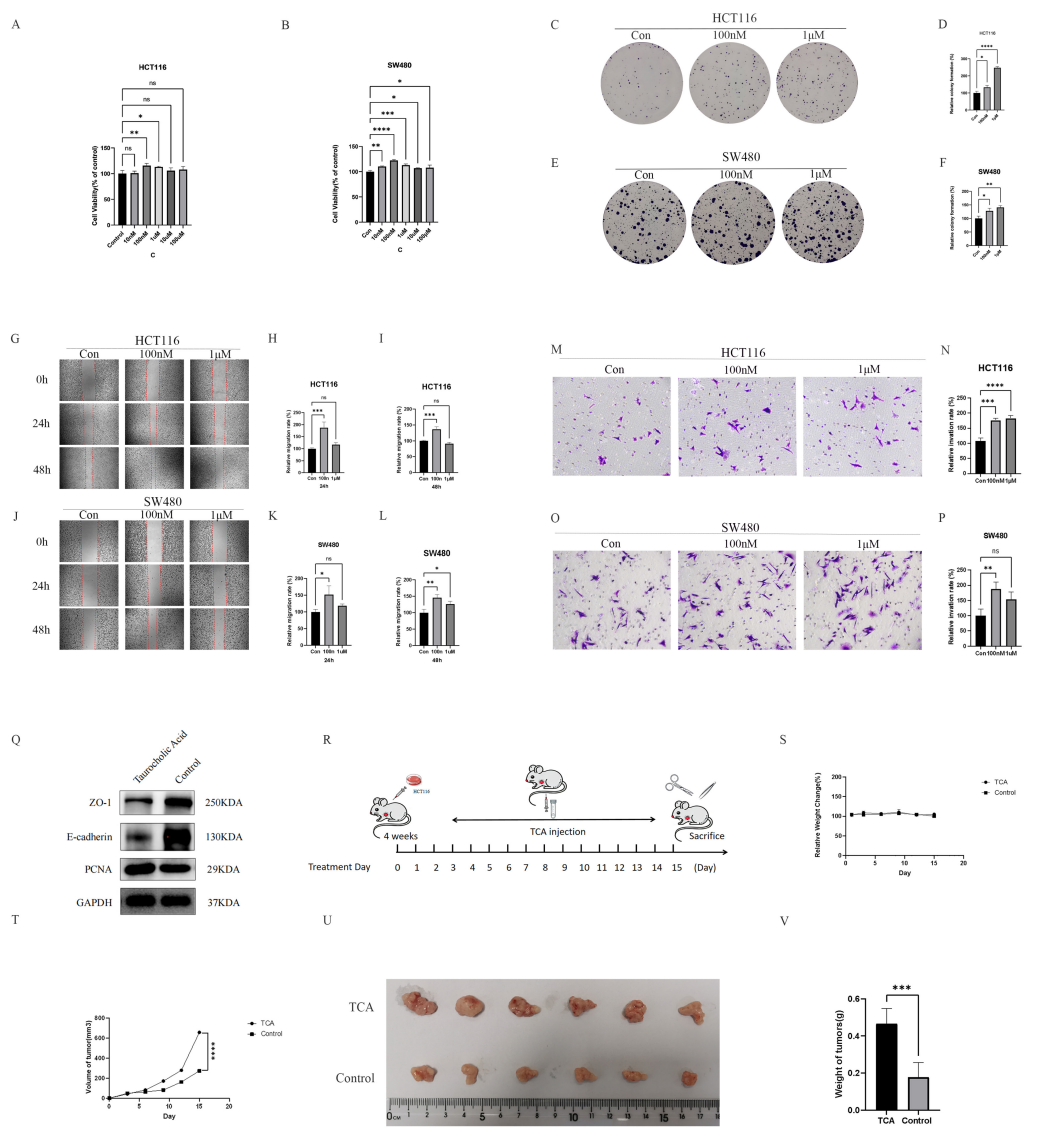
TCA promotes colorectal cancer progression *in vitro* and *in vivo*. (A) TCA significantly increased the viability of HCT116 colorectal cancer cells. (B) TCA significantly increased the viability of SW480 colorectal cancer cells. (C-D) TCA markedly promoted the clonogenic proliferation of HCT116 cells. (E-F) TCA markedly promoted the clonogenic proliferation of SW480 cells. (G-I) Treatment with TCA for 24 h and 48 h significantly enhanced the migration ability of HCT116 cells. (J-L) Treatment with TCA for 24 h and 48 h significantly enhanced the migration ability of SW480 cells. (M-N) Treatment with TCA for 24 h significantly increased the invasion ability of HCT116 cells. (O-P) Treatment with TCA for 24 h significantly increased the invasion ability of SW480 cells. (Q) TCA disrupted intestinal barrier function and enhanced colorectal cancer cell proliferation. (R) *In vivo*, nude mice were administered TCA (125 mg/kg/day, intraperitoneally), and subcutaneous tumors were excised after 15 days. (S) Relative body weight changes in the TCA and control groups during the xenograft experiment. (T) Subcutaneous tumor volume changes in the TCA and control groups during the experiment. (U) Macroscopic images of subcutaneous tumors from the TCA and control groups on day 15. (V) Statistical analysis of tumor weights in the TCA and control groups on day 15.

To evaluate the functional relevance of SD-associated metabolites in CRC progression, two CRC cell lines, HCT116 and SW480, were treated with TCA. Overall, TCA enhanced CRC-associated malignant phenotypes in both cell lines *in vitro*, including increased cell viability, proliferation, migration, and invasion (Figures 6A-P). In parallel, the effects of TCA on the epithelial barrier-related proteins were assessed. Exposure to TCA resulted in reduced protein expression of the tight junction marker, ZO-1, and the adherens junction protein, E-cadherin, in both cell lines compared to that in the untreated controls, indicating compromised epithelial barrier-associated features (Figure 6Q). To further examine the *in vivo* effects of TCA on tumor growth, a subcutaneous xenograft model was established by inoculating HCT116 cells into nude mice. Mice treated with TCA (125 mg/kg/day, intraperitoneal injection) (Figure 6R) exhibited significantly accelerated tumor growth, accompanied by increased tumor volume and weight relative to that in saline-treated controls (Figures 6T-6V). No significant differences in body weight were observed between the two groups throughout the experimental period (Figure 6S). Collectively, these findings support a model in which elevated TCA levels contribute to CRC progression during SD.

### TCA Promotes CRC development via Activation of the MAPK/ERK Signaling Pathway

**Figure 7:**
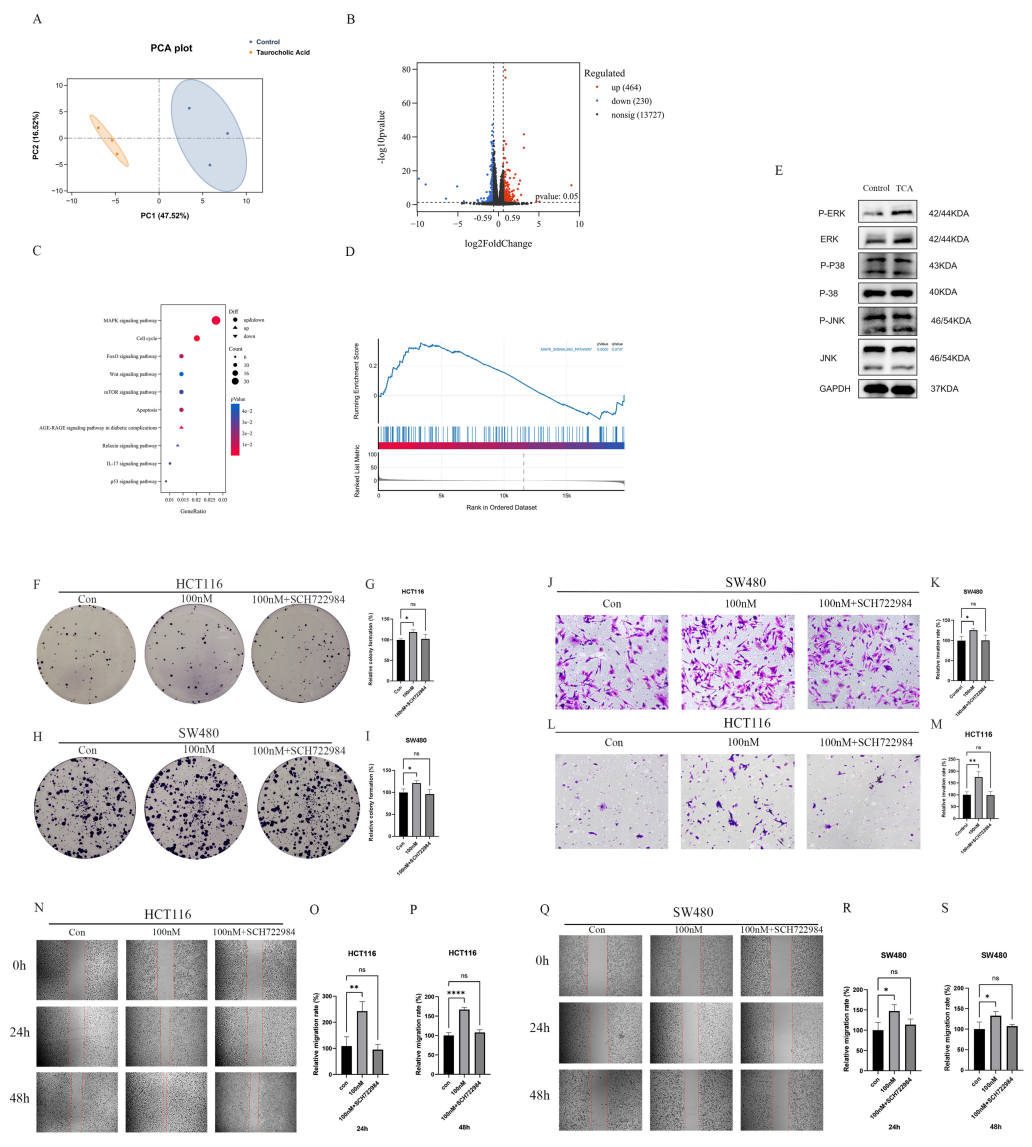
TCA activates the MAPK/ERK signaling pathway to promote colorectal cancer progression. (A) PCA analysis of Control and taurocholic acid (TCA) groups. (B) Volcano plot of differentially expressed genes. (C) KEGG pathway enrichment analysis of differentially expressed genes. (D) GSEA enrichment plot of the MAPK signaling pathway. (E) Western blot showing that TCA may activate the MAPK/ERK signaling pathway. (F-I) TCA promotes proliferation of HCT116 and SW480 cells via the MAPK/ERK signaling pathway. (J-M) TCA enhances the invasive ability of HCT116 and SW480 cells through the MAPK/ERK pathway. (N-S) TCA promotes the migration of HCT116 and SW480 cells through the MAPK/ERK pathway.

To delineate the molecular mechanisms underlying TCA-induced CRC progression, transcriptomic profiling was performed on CRC cells treated with TCA and their corresponding controls. Principal component analysis (PCA) demonstrated a clear separation between the two groups, with TCA-treated samples clustering predominantly on the left and control samples on the right, indicating distinct transcriptional signatures (Figure 7A). Differential expression analysis identified 694 genes that were significantly altered following exposure to 100 nM TCA (|log₂FoldChange| > 1, Padj < 0.05), including 230 upregulated and 464 downregulated genes (Figure 7B). These transcriptional changes suggest broad reprogramming of gene expression in CRC cells in response to TCA.

Pathway enrichment analysis revealed that the MAPK signaling pathway displayed the highest gene ratio among the significantly enriched pathways (Figure 7C). Consistently, gene set enrichment analysis (GSEA) further supported activation of the MAPK signaling cascade in TCA-treated cells (Figure 7D). To validate these transcriptomic findings at the protein level, western blot analysis was performed, which showed a marked increase in phosphorylated extracellular signal-regulated kinase (p-ERK) following TCA treatment, indicating activation of the MAPK/ERK pathway (Figure 7E).

To determine whether MAPK/ERK signaling is required for the pro-tumorigenic effects of TCA, CRC cells were treated with the selective ERK inhibitor, SCH772984. Notably, the pharmacological inhibition of ERK effectively abrogated the TCA-induced enhancement of cell proliferation, migration, and invasion in both HCT116 and SW480 cells (Figures 7F-S). Together, these results demonstrate that the activation of the MAPK/ERK signaling pathway is a key molecular event associated with TCA-mediated CRC development.

### TCA Promotes CRC Development by Activating the MAPK/ERK Pathway via RasGRF1

**Figure 8:**
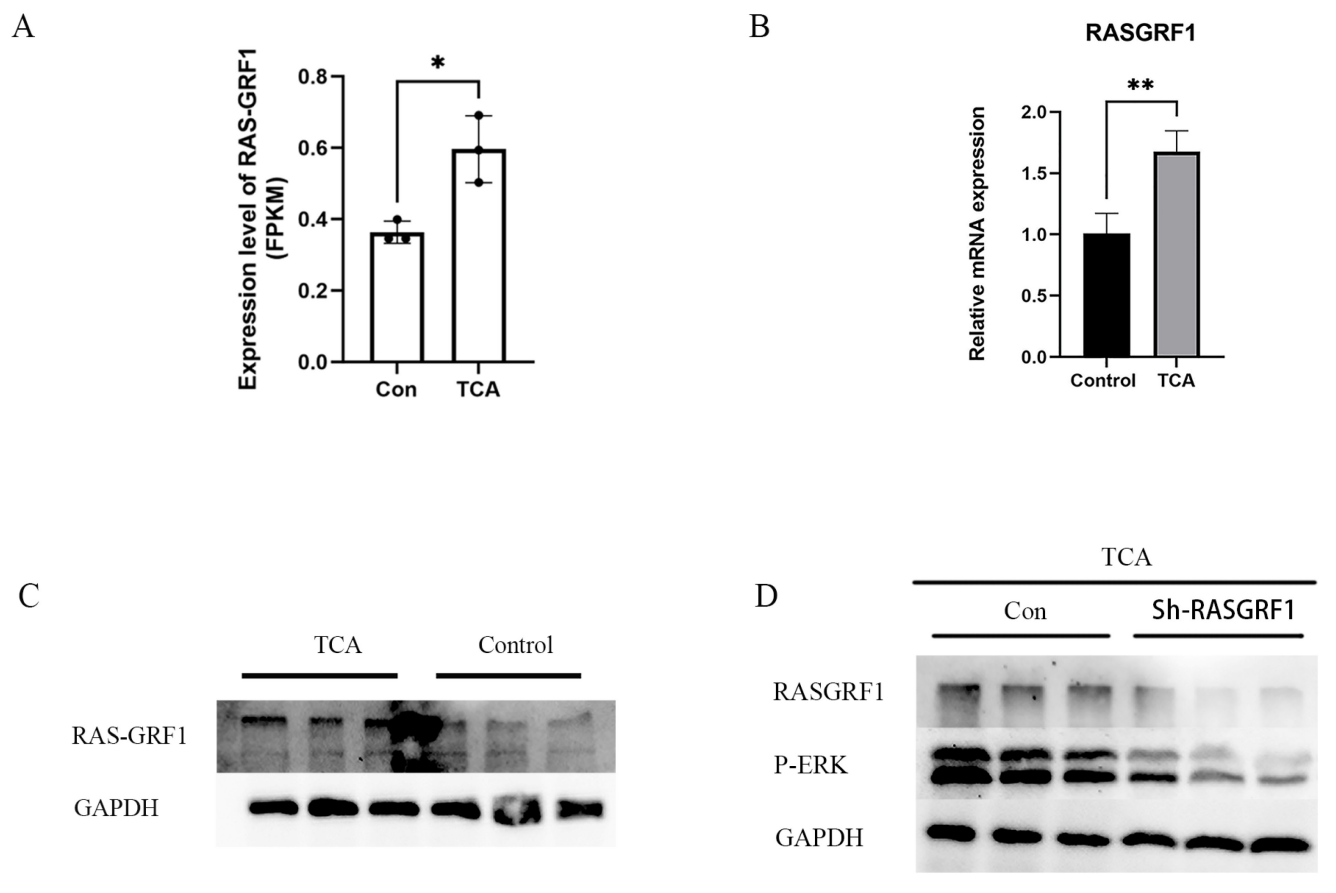
TCA upregulates RasGRF1 expression and activates the MAPK/ERK signaling pathway in CRC cells. (A) Differential expression analysis from transcriptomic data indicating that RasGRF1 is markedly upregulated upon TCA exposure. (B) RT-qPCR validation of TCA-induced *RasGRF1* overexpression in HCT116 colorectal cancer cells. (C) Western blot verification of increased RasGRF1 protein abundance following TCA treatment. (D) Knockdown of *RasGRF1* suppresses TCA-induced ERK phosphorylation in HCT116 CRC cells.

Ras guanine nucleotide-releasing factor 1 (RasGRF1) is a well-established upstream regulator of the MAPK/ERK signaling cascade, functioning through the activation of the Ras-RAF-MEK-ERK axis and contributing to malignant cellular behaviors, including tumor cell proliferation and migration. Previous studies have reported the aberrant upregulation of RasGRF1 in multiple tumor types, with expression levels closely linked to tumor progression. In the present study, transcriptomic analysis revealed a significant increase in RasGRF1 expression in CRC cells after TCA treatment (Figure 8A).

To further assess whether RasGRF1 expression is responsive to TCA exposure, its expression was examined in the CRC cell line HCT116. RT-qPCR analysis confirmed a marked elevation in *RasGRF1* mRNA levels in TCA-treated cells compared to that in controls (Figure 8B). Western blot analysis consistently demonstrated a corresponding increase in RasGRF1 protein levels following TCA treatment (Figure 8C).

To clarify the involvement of RasGRF1 in TCA-induced ERK activation, HCT116 cells were transfected with RasGRF1-targeting short hairpin RNAs (shRNAs) followed by TCA treatment. Silencing RASGRF1 markedly reduced RASGRF1 expression in TCA-treated cells and was accompanied by decreased ERK phosphorylation, as indicated by lower p-ERK levels, while GAPDH remained comparable between groups (Figure 8D). Together, these findings indicate that RasGRF1 is necessary for TCA-mediated activation of ERK signaling and support a RasGRF1-dependent mechanism underlying MAPK/ERK pathway activation in response to TCA.

## Discussion

SD has emerged as an important risk factor for various malignancies, including CRC 9, 13, 19; however, the biological mechanisms linking SD to CRC development remain poorly defined. As a host-co-evolved “second genome,” 20, 21, the gut microbiota plays a central role in CRC progression by modulating metabolic and immune homeostasis 16, 22. Accumulating evidence indicates that lifestyle-related factors—such as smoking and dietary patterns—can influence CRC risk through microbiota-mediated mechanisms 22, 23. In this context, SD represents a prevalent, yet relatively understudied lifestyle disturbance. Therefore, the present study focused on investigating how chronic SD may contribute to CRC progression through the microbiota-metabolism axis, with further emphasis on the potential roles of the candidate metabolite TCA and the RasGRF1/MAPK/ERK signaling pathway in this process.

In the present study, chronic SD markedly increased intestinal adenoma burden and malignant progression in Apc^Min/+^ mice, accompanied by compromised intestinal barrier integrity, enhanced pro-inflammatory responses, and reduced anti-inflammatory signaling. Further analysis revealed that SD markedly altered the composition of gut microbiota. Among the affected taxa, Lactobacillus emerged as a key genus, the abundance of which was significantly reduced in the SD group.

Previous studies demonstrated that Lactobacillus can suppress colorectal tumor growth by enhancing CD8 + T cell-mediated antitumor immunity via indole-3-lactic acid production 24. In addition, both Lactobacillus and Bifidobacterium are reportedly enriched in healthy individuals compared to that in patients with CRC 25, 26. Consistent with these observations, our LEfSe analysis identified Lactobacillus as the most discriminative genus distinguishing the SC and SD groups, with the highest LDA score, thus underscoring its potential importance in SD-associated microbial dysbiosis.

Mechanistically, accumulating evidence indicates that the gut microbiota exhibits circadian rhythmicity, characterized by dynamic oscillations in microbial composition and abundance over a 24-hour cycle 27. Notably, Lactobacillus shows one of the most pronounced circadian fluctuations among commensal bacteria. Therefore, the disruption of sleep-wake cycles may interfere with the circadian regulation of the gut microbiota, contributing to the selective depletion of Lactobacillus observed under SD conditions. Collectively, these findings suggest that SD-induced circadian dysregulation of the gut microbiota leading to Lactobacillus loss may represent an important microbial mechanism linking chronic sleep disturbance to CRC progression.

Beyond alterations in microbial composition, accumulating evidence suggests that gut microbiota-derived metabolites serve as critical functional mediators linking microbial dysbiosis to CRC development 28-30. In line with this concept, the present study demonstrated that chronic SD is accompanied by pronounced remodeling of the fecal metabolomic landscape. Notably, the TCA-related pathway—Taurine and hypotaurine metabolism—was significantly enriched, with TCA being the most prominently upregulated metabolite. Importantly, TCA levels exhibited a strong inverse association with Lactobacillus abundance, suggesting that SD-induced depletion of Lactobacillus may contribute to dysregulated bile acid metabolism and the subsequent accumulation of tumor-promoting metabolites.

TCA has been implicated in the progression of gastrointestinal malignancies via multiple signaling pathways 31-34. However, its role in CRC appears to be context-dependent, with some studies suggesting potential anti-tumor associations under specific therapeutic or metabolic conditions 35, while others have linked elevated TCA to metastatic progression and enhanced malignant phenotypes in CRC 16, 36.

Collectively, these findings suggest that the biological effects of TCA in CRC may vary according to the disease stage, metabolic environment, immune status, and circadian regulation. In addition, the increase in TCA levels reported by Gou *et al*. occurred in the context of a multifactorial therapeutic intervention, suggesting that TCA elevation in this setting may reflect a secondary or compensatory metabolic change rather than a direct tumor-promoting event 35. In contrast, mechanistic evidence from TCA-focused studies, together with its clinical association with metastatic progression and our metabolomic observations, support a potential role for TCA in CRC progression 36.

Consistent with this interpretation, our exogenous TCA supplementation experiments supported the notion that TCA promotes CRC-associated malignant phenotypes. Both *in vitro* and *in vivo* experiments showed that exogenous TCA enhanced CRC cell proliferation, colony-forming capacity, and other malignant features and promoted tumor growth in a subcutaneous xenograft model. Collectively, these findings suggest that TCA may act as a metabolite with tumor-promoting potential in CRC, particularly under chronic SD-associated metabolic dysregulation.

We investigated the molecular mechanisms by which TCA promotes CRC progression. The critical role of the MAPK/ERK signaling pathway in CRC development is well established, with activation of phosphorylated ERK (p-ERK) accelerating tumor progression by inducing cell proliferation, inhibiting apoptosis, and promoting oncogene expression 37, 38. In this study, transcriptomic analysis revealed significant enrichment of the MAPK signaling pathway in TCA-treated CRC cells. Functional experiments further demonstrated that the inhibition of the ERK pathway abolished the pro-tumorigenic effects of TCA, suggesting that TCA promotes CRC progression by activating the MAPK/ERK pathway.

Notably, RasGRF1, as a key upstream regulator of the MAPK/ERK pathway, enhances the activity of this pathway by activating the Ras-RAF-MEK-ERK cascade, thereby exerting pro-tumorigenic effects 39. In this study, both transcriptomic analysis and RT-qPCR showed that *RasGRF1* expression was markedly upregulated in TCA-stimulated HCT116 CRC cells. This was further confirmed by Western blot analysis, which showed elevated RasGRF1 protein levels. Functional validation demonstrated that silencing RasGRF1 significantly suppressed p-ERK activation in TCA-treated CRC cells, confirming that RasGRF1 is an essential mediator of TCA-induced MAPK/ERK pathway activation.

Taken together, our results support a model in which SD promotes CRC development, at least in part, through microbiota-associated TCA elevation and subsequent RasGRF1/MAPK/ERK pathway activation, thereby providing an integrated mechanistic framework linking SD, microbial and metabolic alterations, and oncogenic signaling in CRC.

This study had some limitations. First, while endogenous TCA elevation in SD mice was modest, our exogenous TCA experiments used higher concentrations, primarily for proof-of-concept and mechanistic validation. The intraperitoneal route (125 mg/kg/day) was selected based on previous studies of reproducible systemic exposure 16. Future studies using oral administration, physiologically relevant doses, and direct TCA measurements in serum/feces would better define the quantitative relevance of TCA in SD-associated CRC progression. Second, all experiments were conducted using mouse models. Although the Apc^Min/+^ model has been validated for intestinal tumorigenesis, it primarily reflects genetically driven tumor development and does not fully recapitulate human CRC heterogeneity. Whether SD-associated TCA increases occur in sleep-deprived individuals or in patients with CRC with poor sleep quality remains unknown, warranting clinical and metabolomic validation. Third, pathway dependence has not yet been directly validated *in vivo*. While *in vitro* ERK inhibition and transcriptomic analyses support the involvement of MAPK/ERK in TCA-mediated effects, *in vivo* intervention studies combining TCA with ERK inhibition would strengthen the mechanistic evidence. Fourth, the modified multiple-platform method may introduce stress-related effects beyond those of SD. The observed alterations in the microbiota, inflammation, and tumor progression should be interpreted in the context of both SD and the associated physiological stress. Finally, we did not directly establish whether specific SD-associated microbial alterations, including Lactobacillus depletion, directly regulated TCA accumulation. However, this association requires further functional validation.
